# Editorial: Tumor microenvironment and beyond: imaging systemic immunity with PET

**DOI:** 10.3389/fmed.2024.1535802

**Published:** 2024-12-23

**Authors:** Jelena Levi, Israt S. Alam, Tomomi W. Nobashi

**Affiliations:** ^1^CellSight Technologies, Inc., San Francisco, CA, United States; ^2^Molecular Imaging Program at Stanford, Department of Radiology, School of Medicine, Stanford University, Stanford, CA, United States; ^3^Preemptive Medicine and Lifestyle Related Disease Research Center, Kyoto University Hospital, Kyoto, Japan

**Keywords:** PET imaging of systemic immunity, imaging T cells, imaging biomarkers, imaging checkpoint inhibitor therapy, imaging immunotherapy response, immune-related adverse event

Monoclonal antibodies targeting immune checkpoints, such as CTLA-4, PD-1, and PD-L1, have revolutionized cancer treatment, achieving remarkable long-term remissions in patients with advanced cancers and renewing hope for a cure. The success of immunotherapy, which relies on activating the immune system to target cancer cells rather than killing them directly, has also introduced a more holistic view of cancer and its systemic interactions. The significance of systemic immunity is evident in our own research (1–3), and in this Research Topic we aimed to highlight the role of positron emission tomography (PET)- a whole-body imaging technique- in revealing the complexity and heterogeneity of the immune contexture within the tumor microenvironment (TME) and in assessing systemic immune status and responses to immunotherapy. The Research Topic includes one case report and three reviews that collectively provide an in-depth look at the power and current state of immune-centric PET imaging in immuno-oncology.

Eosinophilic fasciitis, a rare inflammatory condition of the fascia, has been reported as a delayed immune-related adverse event in patients undergoing immune checkpoint inhibitor therapy. Amrane et al. described the first case of eosinophilic fasciitis in a patient with lung cancer treated with stereotactic radiotherapy and pembrolizumab (an anti-PD-1 monoclonal antibody), who achieved complete response after 15 months of treatment. Follow-up [^18^F]FDG PET revealed both diffuse immune thyroiditis, indicative of a good response to therapy (4), and asymptomatic fascial hypermetabolism. Symptoms of eosinophilic fasciitis emerged after additional treatment cycles, eventually prompting the discontinuation of pembrolizumab and initiation of corticosteroid therapy. This case highlights the ability of whole-body PET to detect subclinical off-target immune events, offering valuable insight for early intervention and management of immune-related side effects.

As immune and cancer cells have a shared avidity for glucose, [^18^F]FDG lacks specificity for assessing the immune component within the tumor microenvironment (5). In contrast, PET agents that specifically target immune cells enable the evaluation of complex immunologic processes both within and beyond the TME, and allow for the simultaneous assessment of on- and off-target effects of immunotherapies. In their review, Eertink et al. detailed the essential steps in the development of quantitative imaging biomarkers to assess immune features, framed within the context of the use defined by the FDA's “Biomarkers, Endpoints, and Other Tools (BEST)” initiative. The authors highlighted the value of these imaging biomarkers in revealing intra- and inter-tumoral heterogeneity and supporting early clinical drug development. The review underscores the need for rigorous biological, clinical, and technical validation to ensure biomarker reliability. It also calls for coordinated efforts among PET ligand developers, imaging device manufacturers, pharmaceutical companies, and academic institutions to drive the integration of imaging biomarkers into clinical trials and ultimately into routine clinical practice.

The development of predictive imaging biomarkers is essential to optimize therapy with immune checkpoint inhibitors and for a better understanding of the mechanisms behind varying clinical outcomes. Badenhorst et al. provided a comprehensive overview of the current state of PET tracers developed to assess the PD-1/PD-L1 axis, the most common target of immune checkpoint inhibitor therapy. The authors analyzed the molecular characteristics and binding efficiencies of different classes of tracers, including antibodies, antibody fragments, and peptides, each of which has distinct pharmacokinetic properties, advantages and limitations. Understanding these differences is crucial for optimizing tracer design and advancing the use of imaging biomarkers in personalized immunotherapy.

Complementing the discussion on tracers targeting immune checkpoints, the review by Glazer et al. focused on tracers that monitor T cells—critical players in the antitumor immune response. The authors clearly outlined different approaches to imaging T cells, which include assessment of therapeutic targets, lineage markers and T cell metabolism and function. In describing the preclinical or clinical utility of T cell-targeted imaging agents, the authors highlighted the ability of tracers to assess systemic immunity by noting the importance of tracer accumulation in non-tumor tissues, such as lymph nodes, bone marrow, spleen, and liver. The promising results shown in preclinical settings and small clinical trials underscore the need for larger clinical trials to validate these agents. Ultimately, T cell-based PET imaging may become a vital tool for patient selection, prediction of treatment response, and detection of adverse effects, supporting the advancement of personalized and more effective immunotherapy strategies.

Overall, this special topic sheds light on the potential of PET imaging to fulfill the promise of immunotherapies by enabling a whole-body view of immune dynamics and enhancing our understanding of complex tumor-immune interactions. Larger clinical trials alongside coordinated collaborative efforts will be essential to develop robust biomarkers that enable precision immunotherapy, with the potential to radically improve patient outcomes.

## References

[1] Alam IS Mayer AT Sagiv-Barfi I Wang K Vermesh O Czerwinski DK. Imaging activated T cells predict response to cancer vaccines. J Clin Invest. (2018) 128:2569–80. 10.1172/JCI98509 29596062 PMC5983309

[2] Nobashi TW Mayer AT Xiao Z Chan CT Chaney AM James ML. Whole-body PET imaging of T-cell response to glioblastoma. Clin Cancer Res. (2021) 27:6445–56. 10.1158/1078-0432.CCR-21-1412 34548318 PMC8639766

[3] Levi J Das M Vasanawala MS Behl D Pomper M Forde PM. [(18)F]F-AraG uptake in vertebral bone marrow may predict survival in patients with non-small cell lung cancer treated with anti-PD-(L)1 immunotherapy. J Nucl Med. (2024). 10.2967/jnumed.124.268253 39448270 PMC11619592

[4] Nobashi T Baratto L Reddy SA Srinivas S Toriihara A Hatami N. Predicting response to immunotherapy by evaluating tumors, lymphoid cell-rich organs, and immune-related adverse events using FDG-PET/CT. Clin Nucl Med. (2019) 44:e272–9. 10.1097/RLU.0000000000002453 30688730

[5] Reinfeld BI Madden MZ Wolf MM Chytil A Bader JE Patterson AR. Cell-programmed nutrient partitioning in the tumour microenvironment. Nature. (2021) 593:282–8. 10.1038/s41586-021-03442-1 33828302 PMC8122068

